# The Construction and Evaluation of a Pediatric Emergency Medicine Bootcamp for Graduating Emergency Medicine Residents

**DOI:** 10.1002/aet2.70223

**Published:** 2026-06-21

**Authors:** Michael Hrdy, Regina Toto, Marleny Franco, Ellen Szydlowski, Archana Verma, Pamela Fazzio, Khoon‐Yen Tay, Megan Lavoie, Jill Posner, Anna Weiss, Eva Delgado

**Affiliations:** ^1^ Division of Emergency Medicine Children's Hospital of Philadelphia Philadelphia Pennsylvania USA; ^2^ Department of Clinical Pediatrics University of Pennsylvania Perelman School of Medicine Philadelphia Pennsylvania USA

## Abstract

**Background:**

Clinical rotations during Emergency Medicine (EM) residency do not provide exposure to the full breadth of Pediatric Emergency Medicine (PEM). Caring for critically ill pediatric patients can be challenging, particularly for newly graduated EM physicians. We developed an educational PEM bootcamp for EM senior residents from a diverse group of training programs to address gaps caused by variability in experiences.

**Development Process:**

Following Kern's methodology, we based the bootcamp curriculum on results of a previously published needs assessment and educator perspectives. Graduating residents from the EM programs affiliated with our pediatric emergency department (ED) were invited to a one‐day PEM bootcamp consisting of team‐based simulations, skills stations, and didactics. A retrospective pre‐post survey at the day's end assessed for improvement in participants' perceived preparedness for pediatric emergencies and knowledge of PEM topics taught. Nine months later, participants were invited to a follow‐up survey to assess the impact of the bootcamp on their transition to independent practice.

**Outcomes:**

From 2022 to 2024, 98 residents from 9 distinct programs attended the bootcamp and 84 (86%) completed the end of day survey. There was an increase in the proportion of respondents reporting preparedness to manage pediatric patients, to stabilize pediatric airways, and to perform procedures reviewed. Comments identified respiratory distress management and cardiac disease recognition as lessons to incorporate into future practice. Forty‐three participants from the 2022–2024 cohorts completed follow‐up surveys. Thirty‐seven (86%) reported the bootcamp aided their transition to independent practice.

**Reflective Discussion:**

This annual one‐day PEM bootcamp for our graduating EM residents improved participants' perceived preparedness for pediatric emergencies, bolstered PEM knowledge, and assisted transition to independent practice. This resource‐intensive program is sustained by a dedicated team and continued buy‐in from a wide variety of EM residency programs and adjusted annually in response to participant feedback.

## 1 Need for Innovation

Clinical rotations during Emergency Medicine (EM) residency do not provide exposure to the full breadth of Pediatric Emergency Medicine (PEM). Shifts in the emergency department (ED) are especially unpredictable, with no guarantee that an individual resident will experience the range of pediatric emergencies recommended during training, even if they are exposed to a high volume of children. Focused curricular intervention is needed to fill this educational gap.

## 2 Background

Children make up 20%–25% of emergency care visits in the United States [1]. Most of these children (85%) present to general emergency departments (EDs), where they are cared for by graduates of EM residency programs that require 5 months of dedicated pediatric training during a 36 or 48‐month residency [2, 3]. The volume of pediatric patients and case mix cared for by EM residents differs considerably between residency programs, and exposure to the core pediatric diagnoses as described in the Model of Clinical Practice of EM is not guaranteed [4]. EM residents' experience with critically ill pediatric patients during training is limited [5]. During the first year of practice for EM physicians, critically ill pediatric patients and infants are cited among the most challenging cases to manage [6]. Additionally, EM physicians at many career phases report less comfort with caring for infants and neonates compared with other age groups [7, 8]. In response, some EM residency programs have added or modified clinical rotations that have succeeded in improving EM trainee comfort with infants and children [9, 10]. Further educational initiatives are still needed; however, as reliance on clinical exposure alone is unlikely to generate a sense of preparedness to care for the diverse range of pediatric ages, acuities, and conditions that an EM physician must manage in practice.

## 3 Objectives

To develop a one‐day PEM bootcamp for graduating EM residents with the goals of: (1) improving senior residents' perceived preparedness to care for children, (2) supplementing knowledge gaps in PEM, and (3) positively influencing the graduated residents' transition to attending physician practice.

## 4 Development Process

We designed this curriculum using Kern's six‐step process for curriculum development [11]. This systematic approach enabled us to identify the needs of our learners and to ensure that the educational objectives, teaching strategies, and assessment methods of the bootcamp aligned with those needs. After identifying the educational problem, we conducted a targeted needs assessment by using a modified Delphi process: a panel of experts developed a survey designed to measure perceived preparedness to care for children in three age‐ranges presenting with a variety of pediatric illnesses and injuries [12]. Included in this survey were two free‐text response questions that asked for perspectives on what specific content to focus on and what type of curricula or modality would be best for future educational interventions [13]. We administered this survey from April to June of 2021 to senior EM residents and to recent graduates (1–2 years out of training) from eight EM residency programs. While all of these residency programs use our academic children's hospital as a rotation site for PEM experience, they otherwise differ in primary setting (urban academic vs. community academic vs. community vs. rural), duration of training (3‐ vs. 4‐years), size, and exposure to pediatric patients outside the time spent in our academic, pediatric ED [12, 13]. In addition, in March–April of 2021 we conducted a needs assessment of the eight EM program directors for the residencies targeted by the survey described above. They were advised that their responses were needed to guide a curricular innovation, and they were asked to reflect on In‐Training‐Exam results, procedure logs, and real‐world experience working alongside their residents to help determine if the focus of a new curricular initiative in PEM should focus on high acuity, low occurrence (HALO) scenarios or low stakes, high frequency scenarios, or a combination of both. They were also asked for their perspectives on scheduling and the feasibility of financial support for this initiative.

We operationalized the results of the needs assessments using Kolb's Experiential Learning Theory as a conceptual framework [14]. Kolb's theory recognizes that individual learners engage with educational material in individual ways and describes learning as a cyclical process that encompasses four phases: Concrete Experience, Reflective Observation, Abstract Conceptualization, and Active Experimentation. With these facets of Kolb's theory in mind, we sought to create a curriculum that incorporated a mix of educational formats: didactic lectures, high‐fidelity simulation exercises with debriefing, and hands‐on procedure practice. Procedural and team‐based simulation sessions focused on infants and young children and were designed to cover HALO scenarios as emphasized by the needs assessments [12, 13]. Two topics highlighted in the needs assessment were deemed not amenable to simulation (inborn errors of metabolism and visual diagnosis skills) and therefore were the topics of didactic sessions.

The bootcamp was intentionally planned for the final months of EM residency as just‐in‐time training. We chose this timing as the end of training provokes some anxiety about the realities of solo practice which would drive participation and engagement. This timing was also chosen as the participants in the bootcamp would closely resemble the population surveyed in the targeted needs assessment, which was similarly conducted in spring to capture respondents' perceptions at the end of training (for the senior EM residents) and at the end of the first two attending years (for the graduates). Lastly, timing in late spring also allowed participants from different programs to arrive at the bootcamp having completed a similar amount of training regardless of specific program structure.

Recognizing that educator time, equipment, space, and resident availability are limited, we developed a seven‐hour, one‐day session. We planned to open registration 5 months in advance to allow for schedule requests and the reservation of supplies and rooms. We placed a cap of 35 on the number of participants eligible to attend the bootcamp due to the reality that hands‐on sessions and debriefing are best done in small groups. We planned for seven stations with five simulations, so a cap of 35 would allow for groups of five residents, and each resident would be able to act as lead for a simulation at least once.

## 5 Implementation

We opened registration via email link to an online registration site for the one‐day spring bootcamp starting in winter of 2021, with senior residents from all EM residency programs affiliated with our pediatric emergency department eligible to enroll. The email addresses of program directors, coordinators, and residents were obtained from each program, and notification of how to complete registration was provided in advance by author Dr. Delgado, who has a relationship with these programs as the Director for EM Resident Education and Site Director for Visiting Residents in our ED. There was a registration fee ($100/resident in the first 2 years, then increased to $150/resident thereafter) to cover the cost of facilities and food. The fee was within the proposed budgets of the EM program directors who responded to the needs assessment. The approach to registration and payment varied by program, with some programs registering and paying for their entire senior class and others allowing residents to self‐select and seek reimbursement from programmatic funds as available. This process was repeated yearly, and residents from more EM training programs joined in response to new affiliation agreements. The first PEM bootcamp was held in May of 2022, and it has continued annually.

**FIGURE 1:**
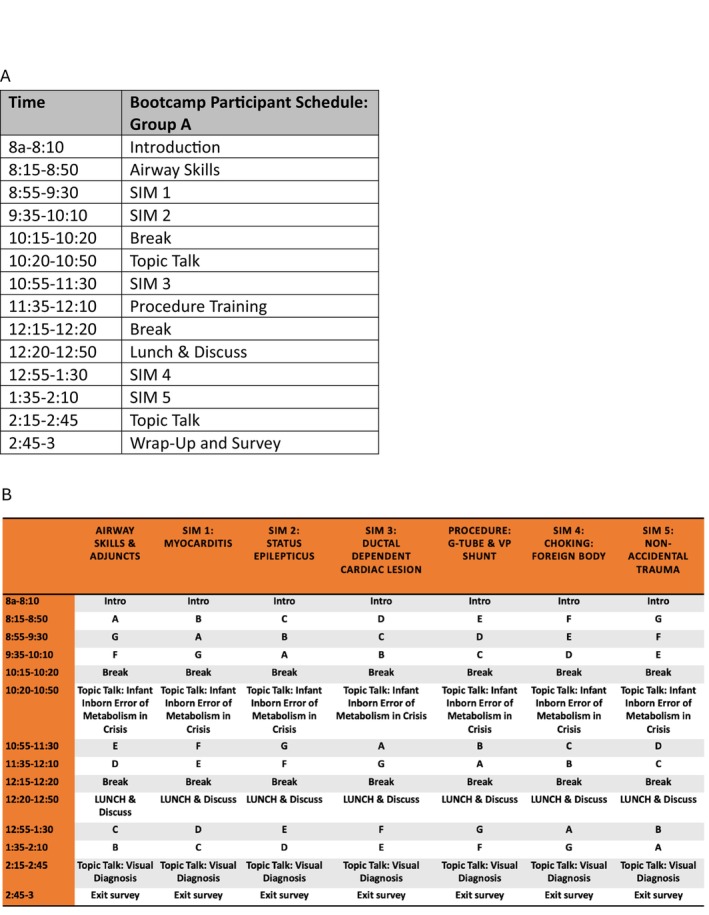
PEM Bootcamp for EM Seniors Sample Schedules: (A) Bootcamp participant schedule representing one individual's experience as a member of Group A. Note that the topic of the simulations and didactics was unknown to the individual until entry into those sessions. (B) Bootcamp director and educator schedule representing overall flow of the participants by group letters, A‐G. Topics of simulations, procedures, and didactics are included here. A version of this schedule without the topics is posted in each room for participants to follow by group letter.

The topics and learning objectives of the stations and discussions were determined by the previously mentioned targeted needs assessments, expert opinion of our simulation faculty who craft simulation for these same EM residents and receive real‐time feedback six times monthly, and in subsequent years, feedback from previous participants. In five 35‐min simulations, we covered recognition and management of: status epilepticus, congenital heart disease, myocarditis, foreign body aspiration, and non‐accidental trauma. We taught pediatric airway management in a hands‐on session each year, since a focus on airway was seen as necessary by senior residents and graduates surveyed in 2021 [13]. Other procedure sessions changed in response to participant feedback and included: ventriculoperitoneal (VP) shunt evaluation, gastrostomy tube replacement, foreign body removal from the ear and nose, umbilical vein catheterization, peripheral intravenous (IV) catheter and intraosseous line placement, and the push‐pull method of administering IV fluids. Large group didactic discussions included sedation, visual diagnosis skills, and the approach to an undifferentiated sick neonate (ultimately diagnosed with an inborn error of metabolism). Residents were assigned to evenly sized groups on arrival and were given nametags labeled with their group letter (A‐G). The course director (Delgado) and administrative assistants provided direction using the schedule template (Figure 1A,B).

The average resources needed for each bootcamp included 25 instructors, 5 simulation mannequins and stations, 3–5 airway heads as well as other airway adjuncts and devices, and 2 models for each chosen procedure. Facilitation, mannikin operation, and instruction were provided by PEM attendings, senior PEM fellows, certified pediatric advanced life support (PALS) instructors, simulation operators, senior respiratory therapists, seasoned PEM nurses, and neonatologists. All simulation facilitators are formally trained in simulation facilitation. Instruction within simulation cases, as well as within procedure and airway management stations, was standardized across multiple facilitators by providing facilitators with focused teaching points for each learning objective as developed by the directors of those stations, each of whom has a background or focus in that area (i.e., senior nursing leadership developed the teaching guide for push‐pull IV fluids).

At the conclusion of the day, all EM resident participants were asked to complete an anonymous end‐of‐day survey that began with retrospective pre‐post Likert‐style questions in which they rated their perceived preparedness to manage pediatric emergencies covered in the session by reflecting on how they felt before the bootcamp relative to how they felt after it was completed. Specifically, they were asked to rate their perceived preparedness on a scale of 1–5 (1 = completely unprepared, 5 = completely prepared) for managing the following: acute illness or injury in pediatric patients, the pediatric procedures reviewed that day, and stabilization of the pediatric airway. The question design was modeled after the 2021 needs assessment of residents and recent graduates from these same training programs [12]. The Likert categories were dichotomized for analysis, with rankings of 4 (moderately prepared) or 5 (completely prepared) combined to represent “prepared”, all other responses were considered “not prepared”. Eleven additional multiple‐choice questions tested knowledge of topics taught, and a retrospective self‐assessment rated participants' perception of their ability to answer these knowledge questions prior to the bootcamp. Many of these questions were previously used in other teaching sessions and therefore had previously undergone pilot testing, and any new questions were pilot tested among the author group and revised as needed. Free text questions asked for one lesson that the participant would incorporate into future practice, and for suggestions for future iterations of the bootcamp. We used McNemar's Chi‐Squared Test with continuity correction to analyze paired quantitative responses and explored free text comments for commonalities among responses.

Following the departure of the participants, the lead educators conducted a debrief of the day, inclusive of perceptions of flow and timing as well as impressions of educator and resident participant experience. One week after the bootcamp, participants received a thank you email from the course director (Delgado) summarizing key lessons and offering an attached file with the full answer key to the knowledge‐based questions from the end‐of‐day survey for their reference.

We distributed a second survey to participants via email 9 months following each bootcamp, in late February of the following year. The email addresses were provided by the residency programs and/or participants themselves, for the specific purpose of conducting a follow‐up survey. In 2023, after the initial email invite, one reminder email was sent 1 week later, and there was no incentive offered. In the following 2 years, both a second reminder email and an incentive were offered to subsequent groups. A largely qualitative assessment grounded in the theory of reflective practice, this anonymous follow‐up survey asked participants to consider: did the bootcamp influence your transition from trainee to attending life? If so, please explain how, and if not, please explain why not. This survey also asked for any suggestions for future bootcamp sessions. The timing of this second survey allowed for participants to graduate residency, transition to attending physician life, and experience caring for children in their EDs as independent physicians prior to responding.

## 6 Outcomes/Evaluation

### 6.1 End of Day Survey Data

From 2022 to 2024, 98 residents from nine different accredited EM programs attended the bootcamp. Most resident participants (58) were from three urban academic EM programs, twenty‐five were from three affiliated community programs, nine were from two community academic programs, and six were from a rural EM residency program. Most resident participants were from three‐year programs, but twenty‐four were from four‐year programs, and thirteen were from five‐year combined programs (EM with Family Medicine or EM with Internal Medicine). For the 2022 session, 32 residents enrolled of 81 invited; for 2023, 44 residents enrolled of 75 invited; and for 2024, 44 residents enrolled of 110 invited. The number of eligible residents changed each year in accordance with program affiliation agreements and variability of program size in some cases. In 2022, 11 residents canceled the day of the bootcamp due to illness and/or shift obligation following a spike in COVID‐19 infections, leaving 21 in attendance. Enrollment in subsequent years was permitted above the cap of 35 in response to this experience, with a plan to start a waitlist once enrollment was over 40. In 2023, 41 residents attended of the 44 enrolled, and in 2024, 36 residents attended of the 44 enrolled. Residents were moved off the waitlist when advanced notice was provided by those who could no longer attend, but cancellations on the day of the bootcamp were not backfilled.

**FIGURE 2:**
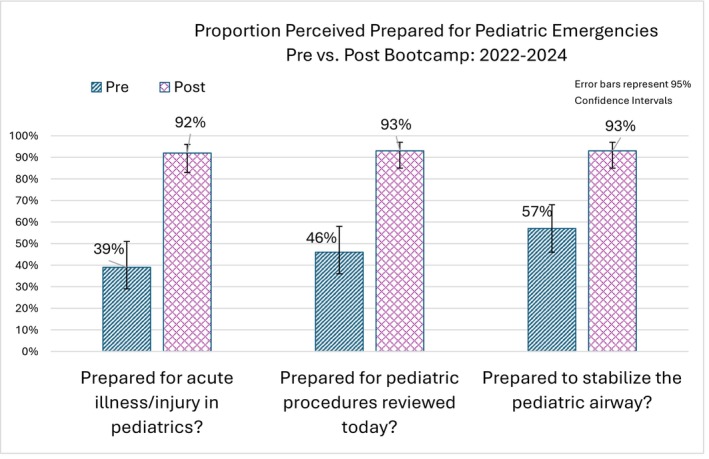
Percentage of Respondents' Perceived Preparedness on the End‐of‐Day Survey. Five‐point Likert‐scale data dichotomized to either Unprepared (1–3) or Prepared (4, 5).

Overall, 84 residents (86%) completed the end‐of‐day survey during this three‐year period. Survey completion was lowest the first year, with a response rate of 62%, resulting in an increased emphasis in subsequent years on the need to complete the survey, and an intentional link between survey completion and validation of parking. The proportion of respondents reporting perceived preparedness in the following domains increased: management of pediatric patients (39% to 92%, +53% [95% CI: 42, 64], *p* < 0.001); stabilization of pediatric airways (57% to 93%, +36% [95% CI: 26, 46], *p* < 0.001); and performance of the procedures reviewed during the bootcamp (46% to 93%, +47% [95% CI: 36, 58], *p* < 0.001) (Figure 2). Results were similar when assessed by individual year, though the number of attendees and the programs they represented varied.

Respondents answered 90% of knowledge questions correctly post‐bootcamp, but there was a range by topic from 69% for myocarditis (identification of exam findings consistent with myocarditis) to 100% for use of push‐pull fluids (recognition of push‐pull fluids as the most efficient way to administer a fluid bolus in a 25 kg child). Respondents' perception of their ability to correctly answer these knowledge questions prior to participating in the bootcamp varied by topic and ranged from 15% (mycoplasma mucositis recognition) to 80% (seizure management).

In free‐text responses, participants commonly noted the identification of respiratory distress and the management and recognition of cardiac disease as lessons they would incorporate into future practice. Respondents also frequently reported finding value in learning how to more effectively utilize pediatric airway equipment. Several also specifically valued learning that respiratory distress can suggest pediatric heart failure, with one sharing that they will remember to “check the liver edge in patients with respiratory distress” as a means of assessing for possible myocarditis or other cardiac compromise. When asked for future suggestions, many respondents offered ideas for procedures to include and others noted that “visual diagnosis was really helpful, actually would love to see more.”

### 6.2 Follow‐Up Survey Data

Forty‐three attending physicians representing the 2022, 2023, and 2024 bootcamp cohorts answered the follow‐up survey sent to them 9 months after completion of the bootcamp (eight from 2022, twenty‐one from 2023, and fourteen from 2024). After few responses were achieved in 2023, the first year the follow‐up survey was done (response rate of 38%), an incentive of $10 gift cards was employed in subsequent years and an additional reminder email was sent (response rate improved to 51% in 2023, though it was 40% in 2024). Most, 86% of respondents (37/43), felt that the bootcamp influenced their transition to attending practice. Thirty‐five percent (15/43) of these physicians reported practicing in a community setting, with the remainder split between academic centers (23%), community affiliates of academic centers (23%), and a combination of different practice settings (19%). Eighty‐four percent (36/43) of these follow‐up survey respondents noted that they care for children in their respective EDs, though with varying frequency: 47% estimated that they treat 1–5 children per week, with 21% treating 11–20 children per week.

Of those 86% who reported that the bootcamp influenced their transition to attending practice, many (26/37, 70%) reported that the bootcamp increased their confidence, comfort, or preparedness to care for children when asked to explain that influence. One respondent shared, “It helped me feel more confident approaching a sick child and helped refresh my approach to common life‐threatening conditions. It also made me realize how much I did not know off the top of my head and inspired me to re‐learn some things.” Attending respondents recognized the importance of the tactile components of the day. As one respondent related, “The hands on, interactive, high‐fidelity sims taught me lessons that I won't forget.” When asked about simulation topics to add to the bootcamp, 28% (12/43) suggested a severe respiratory distress scenario. In response to the question soliciting suggestions for additional procedures to add to the bootcamp, 28% of respondents suggested adding vascular access‐related procedures, including central venous access and umbilical line placement. There were no aspects of the bootcamp that were consistently recommended for removal.

## 7 Reflective Discussion

This annual one‐day PEM bootcamp for EM senior residents offers participants from a diverse group of training programs just‐in‐time exposure to HALO scenarios and procedures to increase their perceived preparedness before graduation. The curriculum was designed with input from the target population, their program leadership, and PEM educators familiar with their training and pediatric exposure, and iterative revisions were implemented based on feedback from participants themselves to ensure continued relevance over time. In addition to its impact on perceived preparedness post‐bootcamp, this innovation also bolstered PEM knowledge and influenced transition to independent practice in a positive way for the majority of participants who responded to our follow‐up survey.

Importantly, the participants in this bootcamp represent the true landscape of EM trainees. Over the past 15– 20 years, there has been an increase in EM residency programs in the United States [15, 16]. Most new programs are 3 years in length, and while a 2020 analysis of EM residents found that 98% were affiliated with programs in urban areas, there has been an increase in community‐based programs [15, 16]. Inherent in this diversity of training site and duration is the variability of pediatric clinical experiences available to each. The Accreditation Council for Graduate Medical Education and the Emergency Medicine Resident's Association agree that all EM residency programs should increase training in pediatric emergency care, but the unpredictable nature of pediatric case exposure for each trainee remains an issue [3, 17]. Our PEM bootcamp addresses this lack of equity in exposure by offering the same curriculum to a diverse group of training programs that is relevant to all, narrowing gaps in experiences across programs due to variability of pediatric access. Given that our participants are representative of the national landscape of EM trainees, it is likely that the construction and adoption of this bootcamp by other educators across the diverse EM residency landscape would yield similar results. Furthermore, these participants, who graduate and then practice in a variety of settings that provide care for children, offered positive feedback on a follow‐up survey, suggesting that this curriculum is also valued by practicing physicians in a variety of post‐graduate practice settings.

We have been able to reproduce the PEM bootcamp for EM senior residents repeatedly at our site by following annual iterations of Kern's model of curriculum development: after reviewing the evaluations (both from the end‐of‐day survey and the follow‐up survey), we identify any new problems, reconsider our goals and objectives, brainstorm any necessary changes to our educational strategies, and then plan for implementation of the next bootcamp [11]. Considerable advanced planning is essential for guaranteeing space for the session, and for ensuring resident, educator, and equipment availability. The administrative burden of room reservation, resident and residency program communication, registration, orientation, and payment is significant, and identification of personnel to complete these steps is essential. Similarly, construction of the educational team and orientation of its members to the goals and objectives is repeated annually to provide updates and clarity. We continue to limit participant group size to maximize each individual's active participation. To address concerns that this cap might exclude interested participants, we doubled the size of the bootcamp in 2026, repeating the same session twice with different participants on different days. Lastly, a program lead or coordinator who is not involved in teaching during the bootcamp allows for someone with situational awareness to direct the movement of groups throughout the day, which is especially important at the end of the day to help encourage participants to join the final debrief and complete the end‐of‐day survey.

Learner feedback and evaluation are essential to the iterative revision process year over year, enabling improvements for subsequent participants. In the feedback obtained at the immediate end of the bootcamp, respondents consistently valued its interactive nature. Therefore, we have continued to use multimodal forms of content delivery—engaging lectures, hands‐on high‐yield procedural and airway skills stations, and team‐based high‐fidelity simulations—allowing learners to manipulate equipment and to engage in real‐time medical decision making. In our follow‐up survey, offered far enough into participants' new post‐residency positions to reasonably expect they will have cared for pediatric patients and reflected on what concepts from the bootcamp were most impactful, respondents offered valuable insights that resulted in concrete changes to the curriculum, particularly a longer visual diagnosis didactic and the inclusion of vascular access in the procedure station.

This curriculum is not without limitations. While our data show a positive reception by respondents, we are limited to subjective outcome measures at the first two levels of the Kirkpatrick framework and are unable to demonstrate actual change in clinical practice. The continued positive reception once the participants are in independent clinical practice supports the conclusion that the curriculum positively affects approach to patient care, but this is also subject to recall and favorability bias. We also cannot know if perception of preparedness changes over time or if knowledge of the topics taught decays after the session since the follow‐up survey did not assess these elements. We expected the impact of the bootcamp to diminish over time, so to acknowledge and address this, we offered participants a few key pearls from each session and the answer key to the knowledge questions via email, though we do not know if they ever used these resources or found them useful. Repetition of other versions of the bootcamp throughout the length of training would likely improve retention and perceptions of preparedness, but this would require a new needs assessment and significantly more resources and support.

Additionally, participation in the bootcamp was not mandatory, though some program directors both paid for their entire senior class and encouraged all of them to attend. It is difficult to know if this biases our results. If the residents who attended felt they needed more supplemental training in pediatrics, there may be a larger effect size of the curriculum for those individuals. At the same time, concluding that those who did not choose to attend were well‐versed in pediatrics may be incorrect, since some EM graduates go on to fellowships in specialties like adult intensive care and so might not feel they need the added pediatric training since it will not impact their future career and not because they already know more about children than their peers.

One particular limitation of the generalizability of this curriculum is how resource‐intensive it is. We intentionally chose to create this bootcamp as a just‐in‐time session for senior EM residents, and this is who we targeted in our needs assessment, since we are unable to offer a curriculum like this for learners at other stages of training as well due to logistics, and we cannot host this event several times per year. Facilitating the program for roughly 30 residents a year requires the ability to perform multiple team‐based simulations and procedural skills stations concurrently with the associated space, simulators, equipment, simulation operators, and facilitators required. Additionally, there is a substantial amount of administrative and logistical planning required prior to the bootcamp day itself. Therefore, smaller programs may need to scale down the curriculum or offer smaller portions of it over several days to fit the programs' individual constraints.

In summary, a one‐day multimodal PEM educational bootcamp for graduating EM residents has been effective in increasing participants' feelings of preparedness to manage acutely ill and injured pediatric patients. In the year following transition to solo practice as attending physicians, the EM graduates surveyed have attested to the bootcamp's positive impact on their abilities and comfort in caring for children. The structure of this bootcamp is feasible and generalizable to a wide variety of residency program types given the diversity of training programs represented in this work.

## Author Contributions

**Eva Delgado:** conceptualization, methodology, investigation, formal analysis, supervision, project administration, writing – original draft, writing – review and editing. **Jill Posner:** conceptualization, methodology, investigation, project administration, writing – original draft. **Ellen Szydlowski:** conceptualization, methodology, investigation, project administration, writing – original draft. **Khoon‐Yen Tay:** investigation, project administration, writing – original draft. **Michael Hrdy:** conceptualization, methodology, data curation, formal analysis, writing – original draft, writing – review and editing. **Marleny Franco:** conceptualization, methodology, investigation, project administration, writing – original draft. **Pamela Fazzio:** investigation, project administration, writing – original draft. **Regina Toto:** investigation, formal analysis, writing – original draft, writing – review and editing. **Archana Verma:** investigation, project administration, writing – original draft. **Anna Weiss:** investigation, project administration, writing – original draft, writing – review and editing. **Megan Lavoie:** conceptualization, methodology, supervision, project administration, writing – original draft.

## Funding

The authors have nothing to report.

## Disclosure

Prior Presentations:

1. Michael Hrdy, Marleny Franco, Khoon‐Yen Tay, Archana Verma, Gonzalez M., Erbayri J., Good G., Ellen Szydlowski, Pamela Fazzio, Regina Toto, Anna Weiss, Posner, P., Megan Lavoie, Eva Delgado, Pediatric Emergency Medicine Bootcamp for Graduating Emergency Medicine Residents. Children's Hospital of Philadelphia Interprofessional Education Conference. Philadelphia, PA, 2023.
2. Eva Delgado, Pediatric Emergency Medicine Bootcamp for Emergency Medicine Senior Residents: From Needs Assessment to Successful Implementation,” Emergency Medicine Education Day, University of Pennsylvania, 2023.
3. Eva Delgado, Michael Hrdy, Marleny Franco, Khoon‐Yen Tay, Archana Verma, Gonzalez, M., Bauman, B., Erbayri, J., Good, G., Ellen Szydlowski, Pamela Fazzio, Regina Toto, Anna Weiss, Posner, J., Megan Lavoie, Curriculum Development: Creation of the PEM Bootcamp for EM Seniors, 7th Annual CHOP Education Symposium: Healthcare Professionals Teaching and Learning Together, Children's Hospital of Philadelphia, 2024.
4. Eva Delgado, Michael Hrdy, Regina Toto, Marleny Franco, Ellen Szydlowski, Bauman, B., Pamela Fazzio, Archana Verma, Tay, K., Megan Lavoie, Posner, J., Anna Weiss, Pediatric Emergency Medicine Bootcamp for Graduating Emergency Medicine Residents: Evaluation of a New Curriculum. Society for Academic Emergency Medicine Conference. Philadelphia, PA, 2025.

## Conflicts of Interest

The authors declare no conflicts of interest.

## Data Availability

The data that support the findings of this study are available from the corresponding author upon reasonable request.
